# Modeling Burns for Pre-Cooled Skin Flame Exposure

**DOI:** 10.3390/ijerph14091024

**Published:** 2017-09-07

**Authors:** Torgrim Log

**Affiliations:** Department of Engineering, Western Norway University of Applied Sciences, 5528 Haugesund, Norway

**Keywords:** flame exposure, wet skin, pre-cooled, thermal injury, numerical modeling

## Abstract

On a television show, a pre-cooled bare-skinned person (TV host) passed through engulfing kerosene flames. The assumption was that a water film should protect him during 0.74 s flame exposure in an environment of 86 kW/m^2^ heat flux. The TV host got light burn inflammation on the back, arms and legs. The present work studies skin temperatures and burn damage integral of such dangerous flame exposure. The skin temperature distribution during water spray pre-cooling, transport to the flames, flame exposure, transport to the water pool, and final water pool cooling is modelled numerically. Details of the temperature development of the skin layers are presented, as well as the associated damage integral. It is shown that 5 °C water spray applied for a 30 s period pre-cooled the skin sufficiently to prevent severe skin injury. Soot marks indicate that the water layer evaporated completely in some areas resulting in skin flame contact. This exposed dry skin directly to the flames contributing significantly to the damage integral. It is further analyzed how higher water temperature, shorter pre-cooling period or longer flame exposure influence the damage integral. It is evident that minor changes in conditions could lead to severe burns and that high heat flux levels at the end of the exposure period are especially dangerous. This flame stunt should never be repeated.

## 1. Background

**Figure 1:**
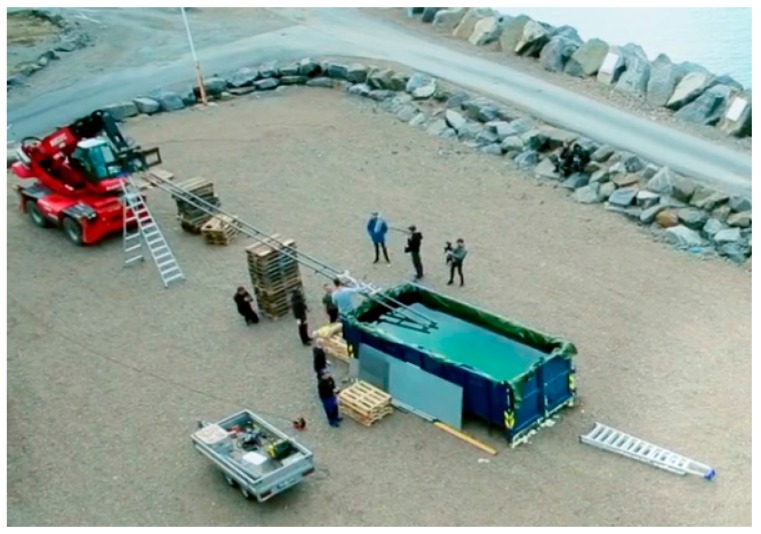
The set-up for the flame exposure (Bulldozer Film Inc., Oslo, Norway). Reproduced with permission.

**Figure 2:**
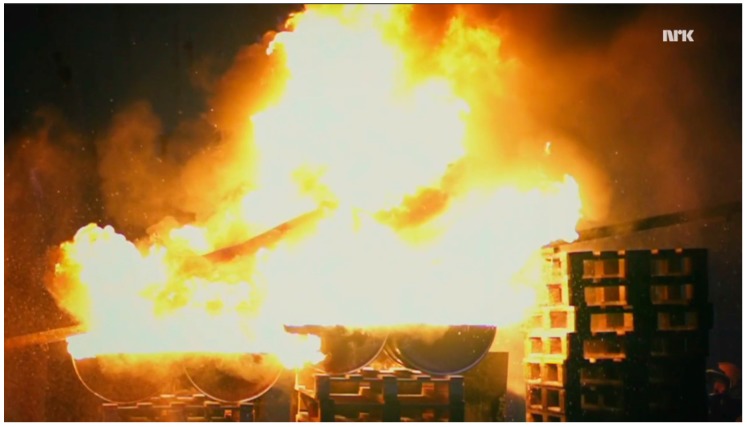
Engulfed in flames en route to the cooling pool (Bulldozer Film Inc., Oslo, Norway). Reproduced with permission.

**Figure 3:**
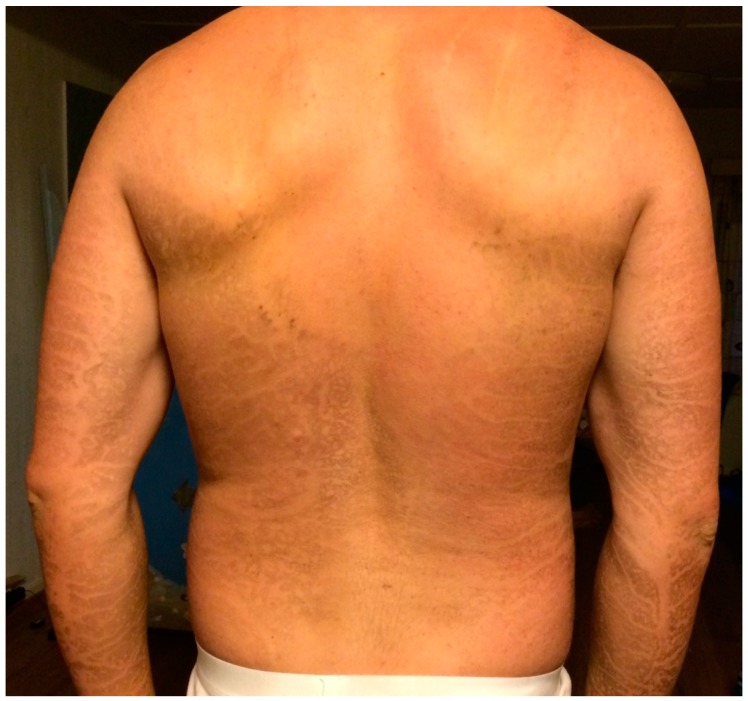
Soot marks and developing inflammation on arms and back (photo: Andreas Wahl. Reproduced with permission).

In the 2016 TV-series *Life on the Line* (*Med livet som innsats*) presented by the Norwegian Broadcasting Corporation, www.nrk.no, physics principles are demonstrated through rather dangerous stunts. The concept is that the physics should protect the TV-host from the dangers he is challenging. Inspired by the folk-physics concept of a wet finger being protected by water evaporation when passed through a candle flame, it was decided to pass a wetted person through large flames. In one of the episodes, named “Grilled Alive”, the TV host, therefore, slid on water-cooled rails through fully-engulfing kerosene flames. A short video for the international audience is available at [1]. An overview photo before the fire was arranged (just to the left of the container pool), is shown in Figure 1. At the start point, the TV-host was sprayed with water at temperature 5 °C for about 30 s in order to be fully wetted before the travel towards the 0.74 s flame exposure. A photo of the flame exposure is shown in Figure 2. Soot marks and developing inflammation after the flame exposure are shown in Figure 3.

The TV host described feeling a stinging pain in the body just before entering the container pool. The inflammation was felt like a sun burn for the next 24–36 h. There was, however, no permanent skin damage. For further details, refer to the video link [1].

## 2. Introduction

After World War II, a series of important studies on the effects of thermal skin injury was published in *The American Journal of Pathology*. These articles included heat transport to, and through, porcine skin, as well as temperature recordings [2]. The importance of time and surface temperature in causing cutaneous burns [3] was also studied, in addition to the pathology and pathogenesis of cutaneous burns on pigs [4]. Most research in fire heat exposure to people lately has been on protective clothing. Full manikin-scale test facilities have, therefore, been built for research and testing [5]. A recent review of this work is given by Zhai and Li [6].

It is generally agreed that a temperature >44 °C will cause burns, with the degree depending on the temperature and exposure time. Recently, more research has been conducted on burns and burn treatment [7,8]. Skin simulators have also been built for studying heat transfer and comparing the developed models to recorded "skin" temperatures during controlled cone calorimeter heat flux exposure [9]. Fu et al. [7] showed that the dermis blood perfusion rate, and the epidermis and dermis conductivity and heat capacity, had little influence on skin damage. Following heating of the skin’s surface, Van de Sompel et al. [10] found that the reduction in Arrhenius damage integrals near the skin’s surface during fast cooling was too small to be physiologically relevant.

Pain receptors are located at a depth of approximately 0.1 mm. The pain temperature threshold is 44.8 °C [11,12]. However, skin injury starts to develop when the skin temperature is greater than 44 °C due to the onset of protein breakdown [13,14]. For hot fluid scalding, other researchers refer to 43 °C as the onset of injury [15]. The resulting skin damage is a function of temperature and time period. Inhalation of hot gases is another major health risk when exposed to flames or hot smoke. Inhalation of air at 1000 °F (538 °C) will instantly result in heat injury to the upper airways above the carina [16]. Flame temperatures, typically close to 1000 °C, therefore, also represent a significant threat for short periods of exposure. An Arrhenius type of damage development is often assumed, i.e., the damage increases considerably with excess temperature.

Fires resulted in 11 million burn injuries hospitalizations in 2004 [17]. In high-income countries, there were 0.14 burn injury hospitalizations per 100,000 persons [18], and it has recently been demonstrated that the risk of hospitalization for fire-related burns increases during extreme cold weather [19]. Burn injuries are, therefore, of major concern worldwide and exposure to flames and hot gases should definitely be avoided.

The present work does, however, investigates voluntary flame exposure of pre-cooled (pre-wetted) skin. Wieczorek and Dembsey [20] present a comprehensive review of the effects of thermal radiation levels and skin injuries. They briefly discuss the benefit of low skin temperatures prior to radiant heat exposure and conclude that that this may help considerably in preventing skin overheating. The concept of pre-cooling is actually used to prevent burns prior to laser dermatology [21]. The only research identified regarding pre-cooled wet human skin exposed to flames is the work by Log [22] where analytical solutions were used to discuss the case studied in the present work. 

The purpose of the present work is to study and analyze dangerous flame exposure of pre-cooled skin by numerically modelling skin heat transfer, skin temperature distribution, and to evaluate skin burn development. The benefit of numerical modelling compared to analytical solutions is that it allows for analyzing the situation all the way from pre-cooling (wetting) through flame exposure and to the water cooling pool. It also allows for modelling changed parameters, such as pre-wetting period, water temperature, exposure time, timing of peak heat fluxes, etc., to study how these different parameters affect burn development.

There are three modelling techniques available [23], i.e., based on the Fourier-type heat transfer equation, wave-type heat transfer equation, or dual-phase lag-type heat transfer equation. The modelling in the present work is based on numerically solving the Fourier-type heat transfer equation, which has been used successfully by other researchers [24,25]. Based on such modelling a very simple scald injury map was developed by Abraham et al. [26] demonstrating the quality of the Fourier-type modelling. In the present work, parameters for the modelling were collected from the TV host, the support crew, and from video footage and the previous study [22].

The paper is unique in analyzing the whole process from pre-cooling through temperature relaxation periods, flame exposure, and post flame exposure water cooling. It is also unique regarding the analysis on early or late peak heat flux exposure on the skin burn damage integral. The paper starts with explaining the background of the case studied (Section 1). Then the introduction describes research on burns and burn modelling (Section 2), followed by a chapter describing the numerical calculations and relevant input parameters (Section 3), a chapter presenting the findings (Section 4), a discussion (Section 5) and a conclusion (Section 6). A strong motivation for completing and publishing this work is to warn about the dangers of such severe flame exposure.

## 3. Modelling Heat Transport and Damage Integral

### 3.1. Heat Transport Modelling

According to Fourier’s law, the heat conducted in a solid is described by a linear relationship between the temperature gradient and the heat flux:

(1) $${\dot{q}}_{k}^{\prime \prime }=-k\cdot \nabla T\;\left(W/m^{2}\right)$$

where k (W/m·K) is the thermal conductivity of the solid, i.e., the skin. The general heat balance for bioheat transfer may be expressed as:

(2) $$\rho C\frac{\partial T}{\partial t}=-\nabla \cdot {\dot{q}}_{k}^{\prime \prime }+W_{b}\rho_{b}C_{b}\left(T_{b}-T\right)+Q_{met}+Q_{ext}\;\left(W/m^{3}\right)$$

where ρ (kg/m^3^) is the skin density, C (J/kg·K) is the skin specific heat, t (s) is the time, $W_{b}$ (m^3^/m^3^ s) is the blood perfusion rate, $\rho_{b}$ (kg/m^3^) is the density of blood, $C_{b}$ (J/kg·K) is the specific heat of blood, $T_{b}$ (K) is the temperature of the supplied blood, $Q_{met}$ (W/m^3^) is the metabolic heat production, and $Q_{ext}$ (W/m^3^) is heat supplied from an external heat source.

In a parametric study, Ng and Chua [27] concluded that the blood perfusion rate did not have much influence on the extent of burns. This confirmed the opinion of Lipkin et al. [28] that about 20 s is needed for the skin to increase the blood flow. Recently, Fu et al. [7] came to a similar conclusion. Since the heat exposure in the present study was very short and represents massive heat transfer compared to any potential blood perfusion and metabolic heat production, only the heat supplied (or withdrawn) by an external source was taken into consideration. This external source is assumed to operate at the surface, and in the present work, a flat body surface area, i.e., the lower back, is studied. This allows for studying heat flow in one dimension, i.e., dependent on the depth (x-dimension) only. 

Assuming thermal properties independent of temperature, the corresponding heat equation below the skin surface is then given by:

(3) $$\frac{\partial T}{\partial t}=\;a\frac{\partial^{2}T}{\partial x^{2\;}}\;\left(K/s\right)$$

where a (m^2^/s) is the thermal diffusivity given by:

(4) $$a=\frac{k}{\rho C}\;\left(m^{2}/s\right)$$

**Figure 4:**
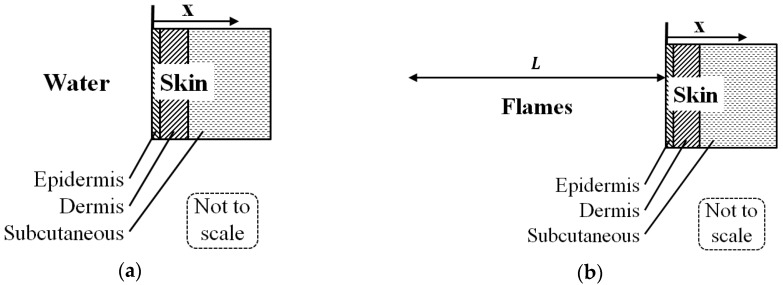
Principle sketch of the one-dimensional heat transfer system: (**a**) During spray and water cooling; and (**b**) during flame exposure. (X indicates the skin depths and L refers to the flame zone.)

The heat transfer model is shown in Figure 4a for the pre-wetting and the container pool cooling and in Figure 4b for the flame exposure. The period of temperature relaxation just prior to and just after the flame exposure, is modeled similarly to the flame exposure without flames and in contact with an adiabatic surface layer, i.e., thermally insulated. This assumption is based on very low heat exchange between the skin and the ambient air compared to water spray cooling and flame exposure.

The heat flux by convection from the skin surface at temperature $T_{S}$ (K) to the water spray at temperature $T_{W}$ (K) may be expressed by:

(5) $${\dot{q}}_{h}^{\prime \prime }=h_{\mathrm{ws}}\cdot \left(T_{S}-T_{W}\right)\;\left(W/m^{2}\right)$$

where $h_{\mathrm{ws}}$ (W/m^2^·K) is the convective heat transfer coefficient estimated to 300 W/m^2^ K by [22]. Based on the 2.9 m flame zone shown in Figure 2 (based on the size of the kerosene pool) and estimated flame properties in [22], i.e., a flame temperature of 950 °C, an optical path length of 0.3 m, and an extinction coefficient of 2.6 1/m, the wet skin on the back was exposed to about 86 kW/m^2^. Based on an analysis including the specific heat of a water layer, water vapor diffusive transport through a transition layer at the body surface, as well as the fact that the water evaporated completely, at most 24 kW/m^2^ was absorbed by heating and evaporating the water film. The readers are referred to [22] for the details of this analysis. The average net heat exposure to the skin was, therefore, estimated to 62 kW/m^2^ as long as there was a water layer present on the skin surface [22].

Solving Equation (3) numerically for the skin layers of the proper thermal properties opens for including all the five phases of heat transfer, i.e., (1) pre-wetting, (2) first temperature relaxation, (3) flame exposure, (4) second temperature relaxation, and (5) water pool cooling. For simplicity, the initial skin temperature was set to 33 °C for all depths x=0 to x=Δ, where Δ (m) is the domain size including the skin layers of Table 1. In order to comply with the external negative or positive heat supply, the skin surface boundary conditions, i.e., at x=0, where the phases 1–5 were set to:

(1) $k\frac{\partial T}{\partial x}=-h_{\mathrm{ws}}\left(T\left(0,t\right)-T_{w}\right)$ for 0 < t ≤ 30.0 s, i.e., pre-wetting phase;
(2) $k\frac{\partial T}{\partial x}=0$ for 30.0 < t ≤ 31.0 s, i.e., first temperature relaxation;
(3) $k\frac{\partial T}{\partial x}=-{\dot{q}}_{\mathrm{net}}^{\prime \prime }$ for 31.0 < t ≤ 31.74 s, i.e., flame exposure;
(4) $k\frac{\partial T}{\partial x}=0$ for 31.74 < t ≤ 33.0 s, i.e., 2nd temperature relaxation; and
(5) $k\frac{\partial T}{\partial x}=-h_{\mathrm{wp}}\left(T\left(0,t\right)-T_{w}\right)$ for 31.5 < t ≤ 40.0 s, water pool cooling.
  where $h_{\mathrm{ws}}$ and $h_{\mathrm{wp}}$ are the heat transfer coefficients for skin to water spray and skin to pool water heat transfer, respectively. The boundary condition for the inner surface is given by the contact with an adiabatic surface, i.e.,
(6) $k\frac{\partial T}{\partial x}=0$ for all t at x=Δ.

The domain size (depth of the skin) must be large enough to limit any influence of the finite dimensions. A depth ∆ > $2\sqrt{at}$ is normally required to minimize the influence of the reflectance heat wave of a limited domain [29], where a (m^2^/s) and t (s) are the thermal diffusivity and time, respectively. The whole subcutaneous layer was, therefore, included in the calculation domain. A comprehensive summary of skin thicknesses and properties as they relate to burns are provided by Johnson et al. [15], referring to trunk epidermis of 42.4 μm. Using the skin properties of Table 1 and a total time period of 40 s gives ∆ ≈ $5.7\sqrt{at}$, i.e., the domain size was sufficiently large to minimize any finite skin depth influence. The water spray temperature was estimated to 5 °C and the convective heat transfer coefficient was estimated to 300 W/m^2^ K in a previous study [22].

In order to achieve numerical stability, the Fourier number must satisfy:

(6) $$Fo=a\cdot \frac{\Delta t}{\Delta x^{2}}<0.5$$

where Δt (s) is the numerical integration time interval and Δx (m) is the layer thickness. A computer program was written in the C++ language to solve the involved numerical equations for the presented boundary conditions. For the numerical integration Δx = 1 μm and Δt = 4 μs were used to comply with Equation (5) ensuring numerical stability for the skin layer of the highest thermal diffusivity, i.e., the dermis layer.

### 3.2. Burn Modelling

The damage index Ω is used to quantify thermal exposure and cell injury due to excessive skin temperatures and protein breakdown, where collagen is one of the main proteins involved [32]:

(7) $$\Omega \left(\tau \right)=\ln\left(\frac{C_{0}}{C_{\tau }}\right)$$

where $C_{0}$ and $C_{\tau }$ represent the number of undamaged cells prior to and after the heat exposure, respectively. A damage index of 0.1 indicates that 90% of the cells are still undamaged after the heat exposure while a damage index of 1.0 indicates that only 36% of the cells are still undamaged. The rate of developing skin damage can be calculated using an Arrhenius-based model developed by Henriques [33]:

(8) $$\frac{\partial \Omega }{\partial t}=P\;\exp\left(-\frac{\Delta E}{\mathrm{RT}}\right)$$

where P (1/s) is the pre-exponential factor (P = 2.185 × 10^124^ 1/s for 44 °C ≤ T ≤ 50 °C and P = 1.823 × 10^51^ 1/s for T > 50 °C) and ΔE (J/mol) is the activation energy for developing skin damage (ΔE = 7.78∙10^5^ J/mol for 44 °C ≤ T ≤ 50 °C and ΔE = 3.27 × 10^5^ J/mol for T > 50 °C [20]. The total damage was integrated over the time interval where the basal layer temperature was ≥44 °C:

(9) $$\Omega =\int_{0}^{t}P\;\exp\left(-\frac{\Delta E}{\mathrm{RT}}\right)\mathrm{dt}$$

Since the damage index is calculated as an integral, it is often referred to as the damage integral. Ye and He [22] report a damage integral Ω = 0.53 at 0.1 mm depth as the limit for first-degree burns, Ω = 1.0 as the limit for second-degree order burns and Ω = 10^4^ for third-degree burns. Comparing the calculated damage integral with these values was used to discuss skin injury for different exposure situations. Numerical integration of Equation (8) was also done in the same C++ program.

It should be noted that there are also another burn classification system dividing burns into superficial burns (equivalent to first-degree burns), superficial partial-thickness burns (extending into the outermost half of the dermal layer), deep-partial thickness burns (passing through the mid-dermal layer and well into the reticular layer), and full-thickness burns (equivalent to third-degree and fourth-degree burns in that they pass completely through the dermis and into the underlying tissue) [34]. The benefit of this second classification system is that it identifies skin burns into categories with different treatment methods. However, since the first system is most recognized within the field of fire safety, this classification system is referred to in the present work.

## 4. Results

**Figure 5:**
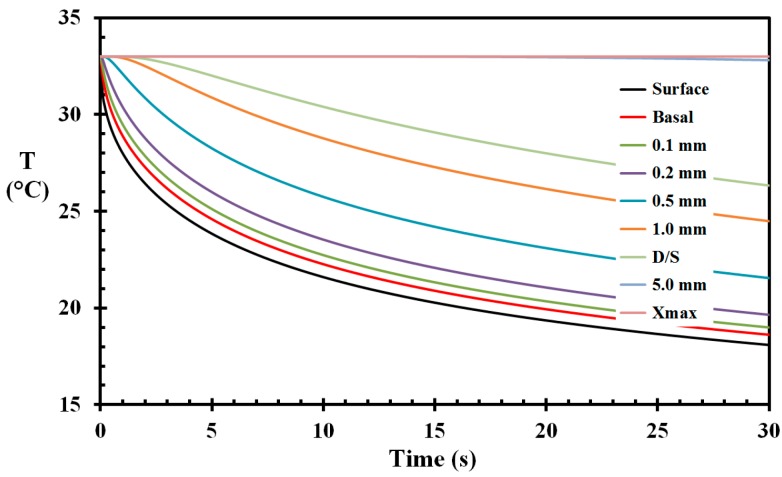
Skin temperature as a function of time during the pre-wetting period for selected skin depths (marked on the figure). D/S represents the dermis–subcutaneous interface.

The temperature development of the skin with the given boundary conditions and the thermal skin properties of Table 1 are shown in Figure 5. It is clearly seen that the pre-cooling reduced the temperatures of the outer skin layers. The epidermis and dermis layer layers were significantly cooled, while only the outer part of the subcutaneous layer was involved in the cooling process. At 5 mm depth, only a very minor reduction in temperature is observed, while at the inner edge of the numerical domain, i.e., at 12 mm depth, a temperature change was first recorded at the fifth decimal after 32 s. This shows that the domain was sufficiently large to prevent any finite domain size influence. The minor temperature drop at 5 mm depth, as seen in Figure 5, indicates that a domain depth of 5–6 mm would indeed have been sufficient for the current modelling. Increasing Δx to 2 μm did not change the results. It was, however, decided to use the initial domain for the succeeding modelling. The only disadvantage was the longer time needed for the calculations (proportional to $1/\Delta x^{2}$).

**Figure 6:**
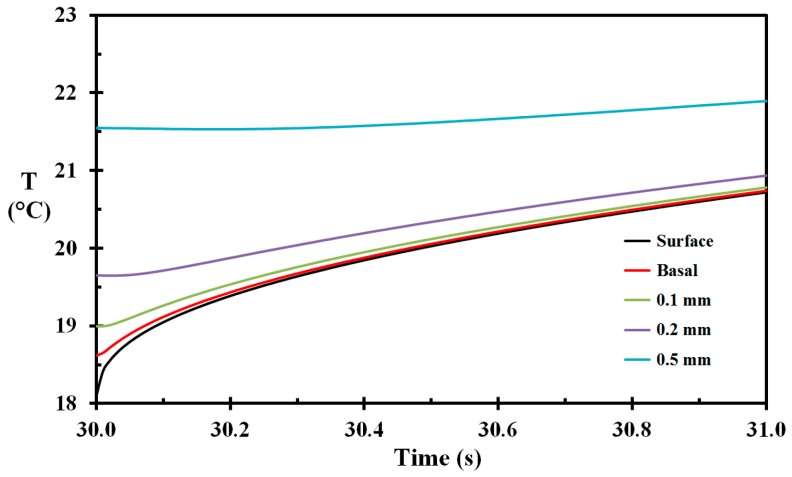
Skin temperature as a function of time during the transport from pre-wetting to the flame exposure for selected skin depths (marked on the figure).

The temperature development during the transport from the spray cooling towards the flame exposure for selected skin depths is shown in Figure 6. It is evident that the temperatures of the outer skin layers do equilibrate some prior to the heat exposure, i.e., the temperatures of the outer 0.1 mm of the skin are quite equal at 31 s, i.e., at the flame entry. During this equilibrating phase, the temperature of the basal layer increases more than two degrees, i.e., from 18.6 °C to 20.7 °C making the skin more susceptible to burn development during the flame exposure.

**Figure 7:**
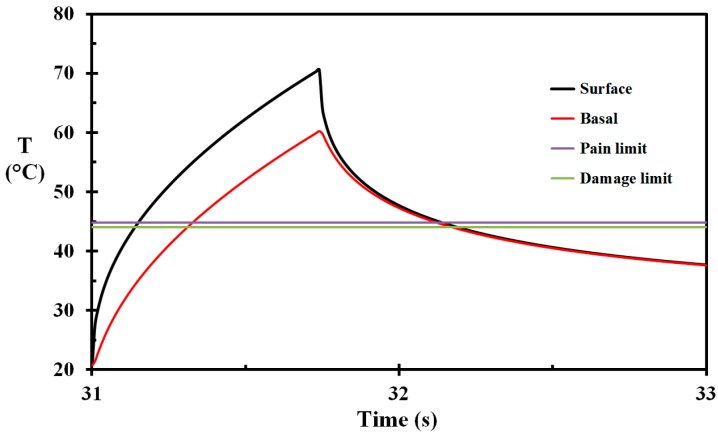
Skin temperature as a function of time during the flame exposure and the temperature relaxation towards the water pool for selected skin depths (marked on the figure). Horizontal lines represent skin damage and pain threshold values.

The heat flux to the wet skin was previously estimated to 86 kW/m^2^ and the heat flux to areas covered by a water film was approximately 62 kW/m^2^ [22]. In the present work, the base case for the skin net heat flux exposure was, therefore, 62 kW/m^2^. The exposure time was 0.74 s. The temperature development during heat exposure and temperature relaxation on the way to the container pool is shown in Figure 7. The temperature of the base layer is above the threshold temperature for developing skin burns, i.e., 44 °C [13,14], in 0.73 s, with 60.2 °C as the peak temperature. The corresponding damage integral at 0.1 mm depth is calculated to 0.13. The pain sensors at 0.1 mm depth reach the pain threshold value, i.e., 44.8 °C, at 31.5 s and stays above this limit for 0.60 s. This is in agreement with the stinging pain experienced by the TV host.

**Figure 8:**
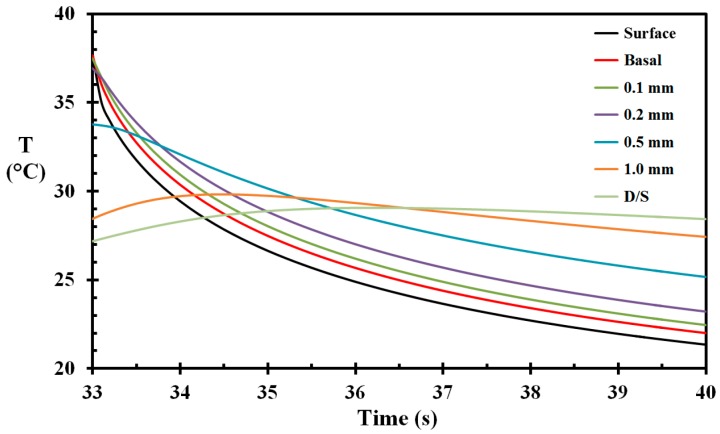
Skin temperature as a function of time during the pool cooling post flame exposure.

According to Wolf and Garner [35] a naked human body immersed in stagnant water is expected to show a heat transfer coefficient between 100 and 200 W/m^2^ K. Given a water thermal conductivity of 0.6 W/m K, this would indicate a heat transfer transition layer of 6 mm and 3 mm, respectively. While the TV-host was moving fast through the water, the transition layer was smaller than this, particularly on the large back surface. If we assume that the transition layer at a certain instant was e.g., 2 mm at the surface we analyzed for burns, the heat transfer coefficient would become 300 W/m^2^ K. This value was used in the present work, though it may have been larger than that, especially in the first second or two (it should be noted that the selected water heat transfer coefficient does not influence the burn development as the temperatures were well below the temperatures associated with burn development while hitting the water surface at 33 s, as seen in Figure 7). The temperature development of the skin during cooling in the water pool is shown in Figure 8. It is clearly seen that the outer skin layers start cooling rather quickly. On the other hand, the deeper layers experience temperature increase while immersed in water. This is due to the previously-initiated flame exposure heat wave still moving inwards at these skin depths, at least in the early phase of the pool cooling. Similar temperature waves expanding diffusively were also observed by Johnson et al. [15], while the surface experienced cooling following hot water scalding.

The net heat flux of 62 kW/m^2^ was the lowest possible heat flux estimated by [22], and the associated damage integral was calculated to 0.13 (Table 2, Case A). Soot deposits, as shown in Figure 3, indicate complete drying on parts of the skin surface, i.e., some parts of the skin surface did experience direct flame contact and heat flux levels of about 86 kW/m^2^. We may, therefore, consider situations where the skin is initially exposed to, e.g., 62 kW/m^2^ and then subsequently exposed to 86 kW/m^2^ during the last part of the 0.74 s exposure time, i.e., $t_{e}$. Assuming that the skin was exposed to 62 kW/m^2^ in e.g., $0.9\cdot t_{e}$ and then to 86 kW/m^2^ in $0.1\cdot t_{e}$, i.e., fully exposed in the last 10% of the flame exposure period (Table 2, Case B), giving a damage integral of 0.25. The opposite case (Table 2, Case C), with exposure to 86 kW/m^2^ in a period of $0.1\cdot t_{e}$ and then 62 kW/m^2^ in the rest $0.9\cdot t_{e}$ of the flame exposure gives less damage integral, i.e., 0.18. The average heat flux, i.e., constant heat flux of 64.4 kW/m^2^ during the exposure period (Case D), gives a damage integral of 0.21.

**Figure 9:**
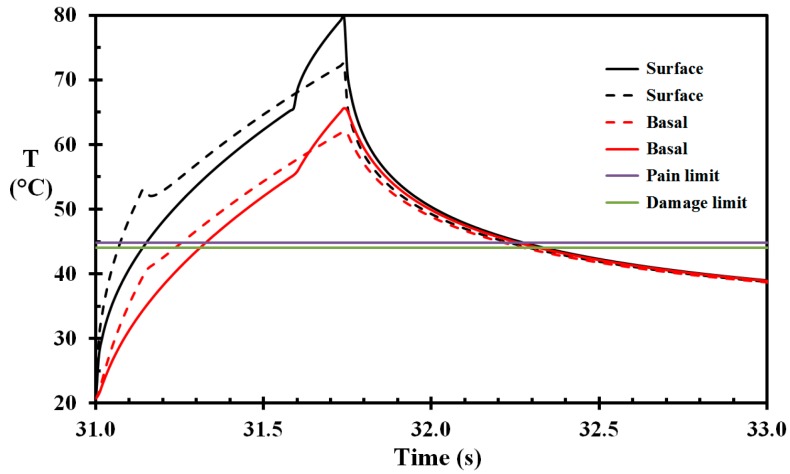
Skin temperature as a function of time when exposed to 62 kW/m^2^ in the first $0.8\cdot t_{e}$ and then to 86 kW/m^2^ in the last $0.2\cdot t_{e}$ of the heat exposure period (Table 2 Case F, solid lines), and opposite (Table 2 Case G, dashed lines).

The temperatures of a similar situation where the skin is, however, fully exposed during either the last 20% or the first 20% of the exposure period (Case E and Case F) are shown in Figure 9. The damage integrals of these cases are 0.55 and 0.26, respectively. For the equivalent average heat flux of 68.4 kW/m^2^ (Case G) the damage integral is 0.49.

An equal amount of heat was supplied in these compared sets of cases (B, C, and D or E, F, and G). If the strong heat flux is supplied early, there is more time for the heat to diffuse deeper into the skin layers, reducing the temperature peak, while a late strong heat supply results in higher peak temperatures. Since the burn damage development according to Equation (8) is exponentially dependent on the temperature, supplying a larger fraction of the total heat late results in higher damage integrals due to the higher peak temperature. This was indeed the case in the real flame exposure, where the unprotected skin was directly exposed after the water layer had drained away or evaporated completely from the skin’s surface.

Compared to the inflammation shown in Figure 3, it may look like the model slightly underestimates the skin burns experienced by the TV host. It is, however, based on a constant heat flux while the water film is evaporating. This may not be correct as the net heat flux to the skin must have been smaller in the start and higher at the end just before the water film evaporated completely and, in particular, when dry skin was directly exposed. It is, however, very challenging to model this, which was outside the scope of the present work. Though not completely realistic, the current model may, however, serve as a tool for studying slightly changed conditions. The 30 s length of the pre-wetting period was based on an interview with the TV host, i.e., it was estimated, not recorded.

Results for different cases such as higher water temperature ($T_{W}$), shorter pre-wetting period ($t_{w}$) and longer flame exposure ($t_{e}$) are listed in Table 2. Case B and Case E are modelled based on the base Case A, but with dry skin exposed to the flames during the last 10% and 20% of the flame exposure. The damage integrals are 0.25 and 0.55, respectively. The heat exposure experienced by the TV host may very well have been within this range.

Comparing reference Case A with Cases H and I, it is seen that the length of the spray cooling period is of importance regarding the skin burns development. Decreasing the spray wetting time from 30 to 10 s increases the damage integral from 0.13 to 0.55, i.e., to first-degree burns. Increasing the water temperature from 5 °C via 15 °C to 25 °C, i.e., Cases A, J, and K, increases the damage integral from 0.13 via 0.56 to 2.38. This would cause very severe burns. An increase in exposure time from 0.74 s (Case B) to 0.80 s (Case L) increases the damage integral from 0.25 to 0.52, i.e., to first-degree burns. A water temperature of 15 °C and a 20 s pre-cooling period combined with a final 10% dry skin exposure gave a damage integral of 1.51, i.e., severe second-degree burns.

The results show that variations in conditions, well within reasonable limits, may cause very severe burns. This illustrates the dangers associated with such flame exposure. Blind copying this flame stunt in a warmer climate where the available water is, e.g., 25 °C would result in very severe burns.

## 5. Discussion

The numerical model, which has also been used by other researchers, e.g., [35,36,37], was shown to give valuable information about skin temperature development and damage integral. The base calculations show that the temperature at 0.1 mm skin depth clearly passed the pain limit, confirming the physiological observation of pain felt in the flame stunt. The model predicts Ω = 0.13, i.e., no first-degree burns on the back skin surface, given the minimum 62 kW/m^2^ net heat flux. This is in contrast to the inflammation described by the TV host and shown in Figure 3, which, according to Lewis et al. [38], fits with epidermal (first degree) burn damage.

If the skin was completely dry and exposed to the estimated 86 kW/m^2^ heat flux for the last 20% of the flame exposure, the model predicts Ω = 0.55, i.e., just above the limit for first-degree skin burns. The model is itself a simplification. The skin was in reality not exposed to a constant average heat flux, i.e., 62 kW/m^2^, during the period until all of the water evaporated. It is more likely that the heat flux increased as the water film heated up and started drying, with a higher heat flux at the end of this period. It was demonstrated that increased heat flux at the end of the exposure period does more harm than a constant equivalent average heat flux throughout the heat exposure period. This is in agreement with the results by Zhai and Li [39] discussing late high heat flux to skin through protective clothing. It should also be noted that the estimated heat flux to the body is probably not even within an accuracy of ±10–20%. The values estimated by [22] are, however, done in accordance with the best practice in fire safety science [21].

In the real flame exposure of the case analyzed in the present work, the skin received some heat flux, i.e., radiation, before entering the flame zone and just after exiting the flame zone. This was not included in the present model. Given these model weaknesses, the model may, however, indicate that without the water film drying completely, first degree burns would not be expected. If the skin dried and was directly exposed for the last 15–16% of the heat exposure period, first-degree burns seems realistic. Though this cannot be verified, it is consistent with soot marks on body parts showing inflammation on the back, under the arms, and under the legs.

In addition to the limitations of the numerical model and assumptions of constant thermal properties of the skin, there were also uncertainties regarding the length of the pre-wetting period. The results are, therefore, only indicative, as are often experienced in case studies where only a limited number of relevant parameters are obtained with some accuracy after an incident of interest. Keeping the uncertainties in mind, the model seems fair in predicting first degree burns in this type of flame exposure, and, when comparing one case to another, assumptions, like the constant thermal properties of the skin, do not significantly influence the results.

An important feature with the modelling is the possibility to analyze slightly changed conditions. It is demonstrated that the length of the pre-wetting period is important. Changing the temperature of the pre-wetting water also has a major influence on the outcomes regarding skin burns. A combination of shorter pre-wetting period and warmer pre-wetting water would likely result in severe second-order burns. This is in agreement with the conclusions by Ng and Chua [27] regarding the initial tissue temperature having a substantial influence on burns development. The results of the present work should, however, be used with care as warmer skin at flame exposure might also result in faster water film evaporation and even longer period of dry skin flame exposure. This could increase the skin damages even more than estimated in the present work. 

Ng and Chua [27] showed that the thickness of the epidermis and dermis has a substantial influence on assessing burn thresholds. It is, therefore, not surprising that the TV host got most of the inflammation on the back which, according to Millington and Wilkinson [31], is among the body parts with the thinnest dermis layer.

The 30-s long pre-wetting period in combination with low water temperature was vital for avoiding severe thermal skin injuries in the case studied in the present work. It gave a temperature margin, as in carefully-tailored tissue cooling prior to dermatology [21]. However, neither the pre-cooling period, nor the water temperature, was planned for in the TV stunt and their fortunately favorable combination was not considered. In the present study, it is clearly demonstrated that a somewhat less favorable set of conditions might have resulted in severe burns. A considerably larger margin should have been chosen or, best of all, such a dangerous flame stunt should never have been undertaken. This was indeed a narrow escape. Blindly copying this flame stunt in warmer climates could certainly result in very serious burns.

## 6. Conclusions

Skin temperature distribution during pre-cooling, transport to the flames, flame exposure, transport to the water pool, and final water pool cooling for a concrete case presented on public TV was modelled numerically. Details of the temperature development in the skin layers were presented, as well as the associated damage integral. It is shown that the water at 5 °C applied for a 30 s period pre-cooled the skin sufficiently to prevent severe skin injury. It is further analyzed how higher water temperature, shorter pre-cooling period or longer flame exposure influence calculated damage integrals. It is shown that minor changes in conditions could lead to severe burns and that high heat flux levels at the end of the exposure period is especially dangerous. This flame stunt should never be repeated.

## Figures and Tables

**Table 1:** Properties of the involved layers (thermal conductivity, k, density, ρ, and specific heat, C, are from [30]. Skin layer thickness estimates for the back are from [31]).

Skin layer	k (W/m K)	ρ (kg/m^3^)	$C_{P}$ (J/kg K)	a (m^2^/s)	Thickness (m)
Epidermis	0.24	1200	3590	5.6 × 10^−8^	43 × 10^−6^
Dermis	0.45	1200	3300	1.1 × 10^−7^	0.0013
Sub cutaneous	0.19	1000	2500	7.6 × 10^−8^	0.01

**Table 2:** Damage integral (Ω) for different variations of water temperature, pre-wetting period, net heat flux, and time of heat exposure.

Case	$T_{W}$ (K)	$t_{w}$ (s)	${\dot{q}}_{\mathrm{net}}^{”}$ (kW/m^2^)	$t_{e}$ (s)	Ω	Comments
A	5	30	62.0	0.74	0.13	Reference case, constant 62 kW/m^2^
B	5	30	64.4 *	0.74	0.25	$0.9\cdot t_{e}$·62 kW/m^2^ and $0.1\cdot t_{e}$·86 kW/m^2^
C	5	30	64.4 *	0.74	0.18	$0.1\cdot t_{e}$·86 kW/m^2^ and $0.9\cdot t_{e}$·62 kW/m^2^
D	5	30	64.4	0.74	0.21	Constant ${\dot{q}}_{net}^{\prime \prime }$= (0.9·62 + 0.1·86) kW/m^2^
E	5	30	68.4 *	0.74	0.55	$0.8\cdot t_{e}$·62 kW/m^2^ and $0.2\cdot t_{e}$·86 kW/m^2^
F	5	30	68.4 *	0.74	0.26	$0.2\cdot t_{e}$·86 kW/m^2^ and $0.8\cdot t_{e}$·62 kW/m^2^
G	5	30	68.4	0.74	0.49	Constant·${\dot{q}}_{net}^{\prime \prime }$ = (0.8·62 + 0.2·86) kW/m^2^
H	5	30	62.0	0.74	0.22	As reference case A, but 20 s pre-wetting
I	5	30	62.0	0.74	0.55	As reference case A, but 10 s pre-wetting
J	15	30	62.0	0.74	0.56	As reference case A, but $T_{W}$ = 15 °C
K	25	30	62.0	0.74	2.38	As reference case A, but $T_{W}$ = 25 °C
L	5	30	64.4 *	0.80	0.52	As case B, but $t_{e}$ = 0.8 s
M	15	20	64.4	0.74	1.51	As case B, but $T_{W}$ = 15 °C and $t_{W}$ = 20 s

* representing the average heat flux.
